# Supplementary material for: Azobenzene Functionalized “T-Type” Poly(Amide Imide)s vs. Guest-Host Systems—A Comparative Study of Structure-Property Relations
Source: Materials (Basel). 2020 Apr 18;13(8):1912. doi: 10.3390/ma13081912 (PMC7215900; doi:10.3390/ma13081912)
Supplement: Supplementary file 1 [file materials-13-01912-s001.pdf]

materials

Article

Azobenzene Functionalized "T-Type" Poly(Amide Imide)s vs. Guest-Host Systems— A Comparative Study of Structure-Property Relations

Karolina Bujak [1], Anna Kozanecka-Szmigiel [2], Ewa Schab-Balcerzak [3] and Jolanta Konieczkowska [3,*]

Figure. S1. X-ray diffraction patterns of investigated azo polymers.
Figure. S2. DTG curves of azo polyimides.
Figure. S3. UV-vis spectra of azo polyimide matrices and their doped analogue for (a) **PAI-1[Az(H)]**, (b) **PAI-3[Az(H)]** in the polymer film.

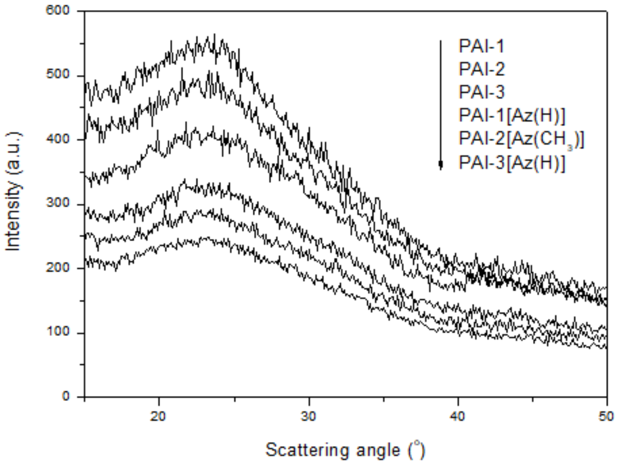

Figure S1. X-ray diffraction patterns of investigated azo polymers.

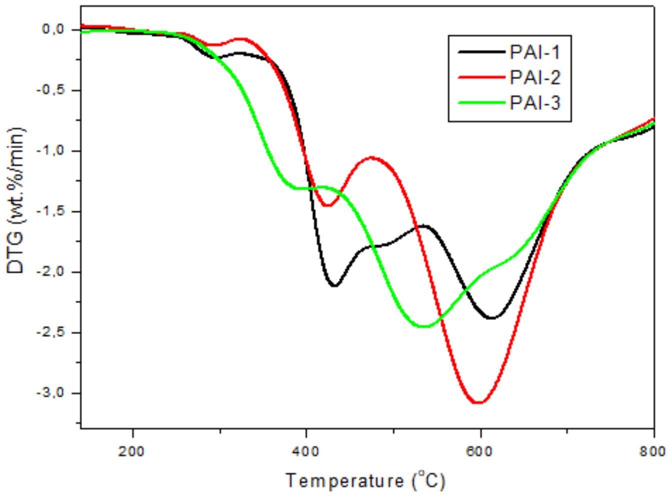

Figure S2. DTG curves of azo polyimides.

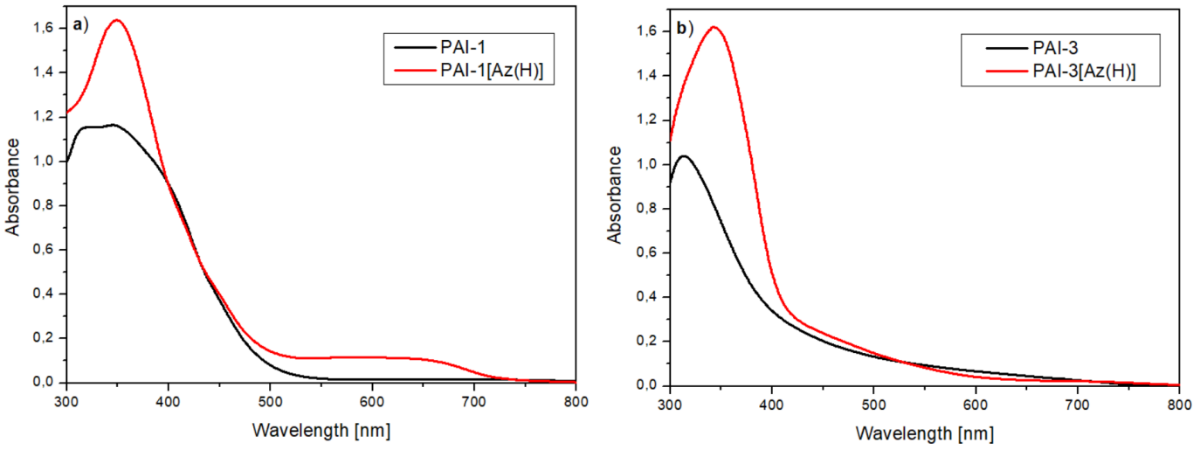

Figure S3. UV-vis spectra of azo polyimide matrices and their doped analogue for (**a**) **PAI-1[Az(H)]**, (**b**) **PAI-3[Az(H)]** in the polymer film.

© 2020 by the authors. Submitted for possible open access publication under the terms and conditions of the Creative Commons Attribution (CC BY) license (http://creativecommons.org/licenses/by/4.0/).
